# Supplementary material for: The implications of using maternity care deserts to measure progress in access to obstetric care: a mixed-integer optimization analysis
Source: BMC Health Serv Res. 2024 May 30;24:682. doi: 10.1186/s12913-024-11135-4 (PMC11137923; doi:10.1186/s12913-024-11135-4)
Supplement: Supplementary file 1 — Supplementary Material 1 [file 12913_2024_11135_MOESM1_ESM.docx]

**Additional File 1**

**Appendix: Mathematical optimization models**

In this appendix, we present our mathematical optimization models used to compare facility expansion policies. This decision-analytic approach provides a way to optimally allocate resources (e.g., facility expansions) across a system in a way that considers constraints (e.g., no more than 4 facilities can be expanded) and decision makers’ objectives (e.g., minimize the number of reproductive-aged (RA) women living in deserts).

**Minimize the number of reproductive-aged women who live in maternity care deserts**

We consider a set of counties $\mathcal{C =}\left\{ 1,2,\ldots,C \right\}$and a set of census block groups $\mathcal{B =}\left\{ 1,2,\ldots,B \right\}$. The population of reproductive-aged women in census block group $b$ is denoted by $p_{b}.$ We use an indicator $\beta_{bc}$ which takes on a value of $1$ if census block group $b$ is within county $c$*.* We use an indicator $q_{c}$ which takes on a value of 1 if county $c$ has no obstetric providers practicing within the county. We use an indicator $x_{c}^{0}$ which takes on a value of 1 if county $c$ has no hospital or birth center providing obstetric care within the county.

For a county to be considered a maternity care desert, it must have no obstetric providers practicing and no hospital or birth center providing obstetric care within the county.

We consider whether a county is a maternity care desert or not using an indicator $d_{c}$, which takes of a value of 1 if a county $c$ is a maternity care desert and 0 otherwise. If a county is a maternity care desert, then all block groups within the county are also maternity care deserts. We indicate if a block group is a maternity care desert using an indicator $d_{b}$,which takes on a value of 1 if census block group $b$ is a desert and 0 otherwise. Thus, the total number of reproductive-aged women who live in maternity care deserts is given by: $\sum_{b=1}^{B} p_{b}d_{b}$.

To consider the impact of policy interventions, we consider the possibility that facilities can be expanded to provide maternity care. We designate *decision variables* $x_{c}\in\left\{ 0,1 \right\}$ such that $x_{c}=1$ indicates county $c$ has a hospital or birth center providing maternity care after infrastructure is expanded. We consider a *constraint* that we can expand at most $F$ facilities to provide maternity care: $\sum_{c=1}^{C} {(x_{c}^{0}- x}_{c})\leq F$. We also consider a set of *constraints* that each county is considered a desert if it has no obstetric providers and no hospital or birth centers within the county: $d_{c}\geq{(q}_{c}+x_{c})-1$. Further, we add another set of *constraints* that each block group is considered a desert only if it is within a county that is a desert: $d_{b}\geq\sum_{c=1}^{C} d_{c}\beta_{bc}$. Finally, we add a set of *constraints* $x_{c}^{0}\geq x_{c}$ which enforce that no county can be downgraded such that they no longer have a hospital or birth center providing obstetric services.

Our optimization model will select the values of the decision variables that satisfy the constraints in order to minimize our objective function. To minimize the total number of reproductive-aged women in maternity care deserts, we will minimize the following objective function:

$$minimize_{x,d}\sum_{b=1}^{B} p_{b}d_{b}$$

Thus, our final optimization model is:

$$minimize_{x,d}\sum_{b=1}^{B} p_{b}d_{b}$$

Subject to:

$$\sum_{c=1}^{C} {(x_{c}^{0}-x}_{c})\leq F$$

$$d_{c}\geq\left( q_{c}+x_{c} \right)-1, \forall c\in\mathcal{C}$$

$$d_{b}\geq\sum_{c=1}^{C} d_{c}\beta_{bc}, \forall b\mathcal{\in B}$$

$$x_{c}^{0}\geq x_{c}, \forall c\in\mathcal{C}$$

$$x_{c}\in\left\{ 0,1 \right\}, \forall c\in\mathcal{C}$$

$$d_{c}\in\left\{ 0,1 \right\}, \forall c\in\mathcal{C}$$

$$d_{b}\in\left\{ 0,1 \right\}, \forall b\mathcal{\in B}$$

We solve this problem for a range of $F$ values ($F$ = 1,2,3,… ) to evaluate how the number of reproductive-aged women living in maternity care deserts changes.

**Minimize the number of women of who live more than 50 miles from CCO services**

We consider a set of census block groups $\mathcal{B =}\left\{ 1,2,\ldots,B \right\}$, and the population of reproductive-aged women in census block group $b$ is denoted by $p_{b}.$ We also consider a set of obstetric facilities $\mathcal{H}=\{1,2,\ldots,H\}$. We use an indicator $x_{h}^{0}$ which takes on a value of 1 if facility $h$ provides CCO services. We use an indicator $\alpha_{bh}$ which takes on a value of 1 if census block group $b$ is within 50 miles of facility *h.*

We consider whether a census block group is further than 50 miles from CCO services using an indicator $d_{b}$, which takes of a value of 1 if a census block group $b$ is further than 50 miles from CCO services and 0 otherwise. Thus, the total number of women who live further than 50 miles from CCO services is given by: $\sum_{i=1}^{B} p_{b}d_{b}$.

To consider the impact of policy interventions, we allow lower-level facilities that do not provide CCO services to be upgraded to provide CCO services. We consider *decision variables* $x_{h}\in\left\{ 0,1 \right\}$ such that $x_{h}=1$ indicates that facility $h$ provides CCO services after upgrades. We consider a *constraint* that we can upgrade at most $F$ facilities to provide critical care obstetric services: $\sum_{h=1}^{H} {(x}_{h}-x_{h}^{0})\leq F$. Further, we add a set of constraints $d_{b}\geq1-\sum_{h=1}^{H} \alpha_{ch}x_{h}$ which enforce that a census block group lacks access if it is further than 50 miles from its nearest obstetric facility offering CCO services. Finally, we add a set of constraints $x_{h}\geq x_{h}^{0}$ which enforce that no facilities can be downgraded such that they no longer provide CCO services.

Our optimization model will select the values of the decision variables that satisfy the constraints in order to minimize our objective function. To minimize the total number of reproductive-aged women who live further than 50 miles to CCO services, we will minimize the following objective function:

$$minimize_{x,d}\sum_{b=1}^{B} p_{b}d_{b}$$

Thus, our final optimization model is:

$$minimize_{x,d}\sum_{b=1}^{B} p_{b}d_{b}$$

Subject to:

$$\sum_{h=1}^{H} {(x}_{h}-x_{h}^{0})\leq F$$

$$d_{b}\geq1-\sum_{h=1}^{H} \alpha_{bh}x_{h}, \forall b\mathcal{\in B}$$

$$x_{h}\geq x_{h}^{0} , \forall h\in\mathcal{H}$$

$$x_{h}\in\left\{ 0,1 \right\}, \forall h\in\mathcal{H}$$

$$d_{b}\in\left\{ 0,1 \right\}, \forall b\mathcal{\in B}$$

We solve this problem for a range of $F$ values.
